# Potentially burdensome end-of-life care for colorectal cancer decedents: A retrospective cohort study

**DOI:** 10.1017/S1478951526103095

**Published:** 2026-07-09

**Authors:** Gifty Varghese, Tolesa Okuba, Geoffrey Delaney, Gaston Arnolda, Winston Liauw, Reidar Lystad, Reema Harrison, Jeffrey Braithwaite, Rebecca Mitchell

**Affiliations:** 1 Australian Institute of Health Innovation, Faculty of Medicine, Health and Human Sciences, Macquarie University, Sydney, Australia; 2 Sydney Partnership for Health Education Research and Enterprise, Australia; 3 Liverpool Cancer Therapy Centre, Australia; 4 University of New South Wales School of Clinical Medicine, Australia

**Keywords:** Colorectal cancer, end-of-life care, health services research, palliative care, retrospective cohort study

## Abstract

**Objectives:**

Colorectal cancer is a leading cause of cancer mortality in Australia, with many patients requiring complex end-of-life care. Evidence of potentially burdensome end-of-life care specific to colorectal cancer populations in hospital settings is limited. This study aimed to examine factors associated with indicators of potentially burdensome end-of-life care among people who died from colorectal cancer and received hospital-based care in New South Wales, Australia.

**Methods:**

A retrospective population-based cohort study was conducted using linked data from the NSW Cancer Registry, hospital, and mortality records (2014–2019). Adults aged ≥20 years whose underlying cause of death was colorectal cancer and who were hospitalized in their final year of life were included. Multivariable logistic regression models examined associations between patient demographics and all indicators. Multinominal logistic regression examined predictors of a composite indicator comprising 4 indicators of potentially burdensome end-of-life care.

**Results:**

Of 9,476 colorectal cancer decedents, 15.7% died in acute care. Within the last 30 days of life, 13.1% had >1 emergency department presentation, 9.2% had >1 hospital admission, and 3.1% had an intensive care unit admission. The composite indicator identified 71.3% of patients with no indicators, 18.9% with 1, and 9.8% with ≥2 indicators of potentially burdensome end-of-life care. Higher odds of potentially burdensome end-of-life care were observed among people who smoke, people living in rural locations, who had a lower socioeconomic status, a prior cancer diagnosis, or their final admission was to a private hospital. Females, people with comorbidities, and people who had a longer survival duration, had lower odds of potentially burdensome end-of-life care.

**Significance of results:**

Findings highlight socioeconomic and system-level disparities that may inform policy and clinical strategies to improve equitable, patient-centered end-of-life care.

## Introduction

Colorectal cancer is the second leading cause of cancer-related mortality worldwide, with an estimated 1.9 million new cases and 904,000 deaths in 2022 (Bray et al. 2024). In Australia, colorectal cancer is responsible for nearly 5,300 deaths each year (Cancer Australia 2024). Advances in cancer treatment have extended survival duration, while simultaneously highlighting the importance of symptom control and better quality of life near the end-of-life (Costi et al. 2014). Yet evidence consistently indicates that patients continue to receive potentially burdensome care at the end-of-life (Hu et al. 2014; Mitchell et al. 2024).

Indicators of potentially burdensome end-of-life care, such as multiple hospitalizations, intensive care unit (ICU) admissions, emergency department (ED) visits, or chemotherapy in the final week of life, are important as they may reflect care that offers limited clinical benefit, can negatively impact quality of life, and is often expensive and in limited supply. Despite growing attention, limited Australian evidence exists that examines indicators of potentially burdensome care at end-of-life, particularly for colorectal cancer populations (Mitchell et al. 2024). Previous studies have examined mixed cancer cohorts (Abdel-Razeq et al. 2019; Allende-Pérez et al. 2024; Boddaert et al. 2024), but colorectal cancer-specific studies on potentially burdensome end-of-life care are limited, despite the distinct disease trajectory of colorectal cancer (Hu et al. 2014). Palliative care is defined as a person-centered approach that aims to improve the quality of life and alleviate suffering for patients with serious illnesses (Rome et al. 2011). High-quality palliative care is essential for reducing unnecessary hospitalizations or interventions and aligning care with patient values and preferences (Rome et al. 2011).

Understanding how patient, disease, and health system characteristics could influence potentially burdensome end-of-life care within the Australian context is critical to enhancing the quality of end-of-life care delivery for colorectal cancer populations (Mitchell et al. 2024). This study aims to examine factors associated with indicators of potentially burdensome end-of-life care among people who died from colorectal cancer and received hospital-based care in New South Wales (NSW), Australia.

## Methods

### Study design

A retrospective cohort study of people who had an underlying cause of death from colorectal cancer in NSW, Australia, during 2014–2019. Mortality data were linked to hospital and cancer registry records for 12 months prior to the date of death. The reporting methods were guided by the STROBE checklist (Cuschieri 2019).

### Data sources and record linkage

Data were obtained from the NSW Cancer Registry, ED presentations and hospital admissions in NSW, nonadmitted patient records, and the NSW Registry of Births, Deaths and Marriages and the Cause of Death Unit Record File (COD-URF). Extracts from these datasets were linked by the Centre for Health Record Linkage (CHeReL) using probabilistic linkage. Probabilistic linkage uses multiple identifiers to estimate whether records from different datasets belong to the same individual when no unique identifier exists (Blake et al. 2021). Record groups with linkage probabilities between 0.25 and 0.75 underwent clerical review to ensure accuracy.

Hospital records were sourced for nonadmitted patient services, ED presentations, and hospital admissions across NSW. The nonadmitted patient records identified chemotherapy and radiotherapy services at public hospitals. These data were only available from 2016 to 2019 and included information on patient demographics, service type, mode of contact (e.g. in-person, videoconference), and service provider. Records without any client contact were excluded.

ED presentations to public hospitals in NSW included details on arrival and departure times, visit type, and separation outcome. Hospital admissions across all public and private hospitals in NSW contained information on principal and additional diagnoses, clinical procedures, and separation type, including transfer to another facility or death. Hospital admission diagnoses were classified according to the International Classification of Diseases, 10th Revision, Australian Modification (ICD-10-AM). Mortality data included date of death and up to 20 antecedent underlying causes of death classified using the International Classification of Diseases, 10th Revision (ICD-10).

The NSW Cancer Registry contains notifications of people with cancer in NSW (except for nonmelanoma skin cancer) and was used to identify prior history of cancer, date of diagnosis, spread at diagnosis, and place of death. NSW Cancer Registry data were provided from 1972 to 2019.

### Case inclusion criteria

Adults aged ≥20 years whose underlying cause of death was colorectal cancer (ICD-10: C18–C20) in the COD-URF in NSW, Australia, between 1 January 2014 and 31 December 2019 were included. Patients were excluded if they died within 30 days of diagnosis or had a primary cause of death not attributed to colorectal cancer.

### Residents of aged care and place of death

Patients living in residential aged care during their final hospital admission were identified using hospital record information such as separation mode, source of referral, and financial class. Deaths occurring in acute care were identified using admission and separation dates, date of death, and separation mode recorded in hospital admission data. Deaths in the ED were identified using separation mode. For individuals who did not use hospital services, place of death was taken from the NSW Cancer Registry.

### Palliative or hospice care

Palliative or hospice care was identified using any data items in hospital admission records that indicated palliative or hospice care (i.e. episode of care type, service-related group, unit type on admission, peer-group, facility type, separation mode) or an additional diagnosis in up to 50 diagnosis codes of palliative care (ICD-10-AM: Z51.5).

### Intensive care unit admissions and mechanical ventilation

Hospital admission records identify hours in an ICU, which were categorized as ICU admission (Y/N). Hours on mechanical ventilation are recorded in hospitalization records and were categorized as mechanical ventilation (Y/N).

### End-of-life care indicators

The indicators of potentially burdensome end-of-life care for colorectal cancer patients were identified from the literature and where the indicators could be identified in population-based administrative health data collections in NSW (Table 1). A composite measure of potentially burdensome end-of-life care was defined as having at least one of the following: >1 ED presentation, >1 hospital admission, ≥1 ICU admission, or death in acute care in the last 30 days of life (Earle et al. 2003; Hu et al. 2014; Mitchell et al. 2024). The composite measure was categorized as: no indicator, 1 indicator, or 2 or more indicators of potentially burdensome end-of-life care.

**Table 1.** Indicators of potentially burdensome end-of-life care that could be identified in population-based administrative health data collections Table 1 long description.

Potentially burdensome EOLC indicators	
>1 ED visit in the last 30 days of life	Earle et al. (2004); Earle et al. (2008); Ho et al. (2011); Hu et al. (2014); Jang et al. (2015); Bähler et al. (2018)
>1 hospitalization in the last 30 days of life (excluding palliative or hospice care)	Earle et al. (2008); Ho et al. (2011); Hu et al. (2014); Jang et al. (2015); Bähler et al. (2018)
≥1 ICU admission in the last 30 days of life	Earle et al. (2004); Earle et al. (2008); Ho et al. (2011); Hu et al. (2014); Jang et al. (2015); Chiang and Kao (2017); Ersek et al. (2017); Bähler et al. (2018); Ní Chróinín et al. (2018)
Place of death at EOL was in acute care (excluding palliative or hospice care)	Earle et al. (2004); Bähler et al. (2018); Ní Chróinín et al. (2018)
>14 days in hospital in the last 30 days of life (excluding palliative or hospice care)	Earle et al. (2004)
Mechanical ventilation in the last 30 days of life	Chiang and Kao (2017); Ersek et al. (2017)
Radiotherapy in the last 30 days of life	Robausch et al. (2021)
Last dose IV chemotherapy in the last 7 days of life	Earle et al. (2004); Earle et al. (2008); Ho et al. (2011); Hu et al. (2014); Jang et al. (2015); Bähler et al. (2018)
Last dose IV chemotherapy in the last 14 days of life	Earle et al. (2004); Hu et al. (2014)

### Identification of comorbidities

The Charlson comorbidity index (Quan et al. 2011) was used to identify comorbidities using up to 50 diagnosis classifications in hospitalization records using ICD-10-AM codes with a 1-year lookback from the date of death. Charlson comorbidities excluding malignancies were categorized as 0, 1, or ≥2 comorbidities. Comorbid conditions related to mental health (ICD-10-AM: F00–F99) and tobacco use (ICD-10-AM: F17.0–F17.9, P04.2, T65.2, Z58.7, Z71.6, Z72.0, Z81.2, Z86.43) were also identified using hospital diagnosis records.

### Socioeconomic status and geographic location

Socioeconomic disadvantage was measured using the index of relative socioeconomic disadvantage from Socio-Economic Indexes for Areas (ABS 2011a) and Statistical Area Level 2 (SA2) of residence in hospital or NSW Cancer Registry records. Scores were grouped into quintiles (1 = most disadvantaged to 5 = least disadvantaged). The quintiles are derived from Australia’s population census using information including education, employment, occupation, and income. Geographic location was determined using the Australian Statistical Geography Standard Remoteness Areas (ABS 2011b) or SA2 of residence in hospital or NSW Cancer Registry records. These were categorized as urban (major cities) and rural (inner regional, outer regional, remote, very remote) based on distance to service centers.

### Chemotherapy and radiotherapy

Chemotherapy administered in the hospital was identified from hospital records using a principal diagnosis of cancer (ICD-10-AM: C00–C96, D45–D47.5) and procedure block codes 1920 (“administration of pharmacotherapy”) or 1922 (“other procedures related to pharmacotherapy”), or AR-DRG R63Z (“chemotherapy”). Nonadmitted patient data were also used to identify outpatient chemotherapy services, as was radiotherapy, using service types and service classifications.

### Data management and analysis

Descriptive statistics summarized cohort characteristics, and chi-square tests of independence compared categorical variables. Logistic regression examined factors associated with each indicator, and multinomial logistic regression examined predictors of the composite measure, of potentially burdensome end-of-life care. Variables with *p* ≤ 0.20 in univariate analyses were included in backward stepwise models (significance at *p* < 0.05). Backward stepwise modeling sequentially removes nonsignificant variables to identify the most important predictors (Chowdhury and Turin 2020). Odds ratios (OR) and 95% confidence intervals (CIs) were calculated. Analyses were conducted in Stata v18 (StataCorp 2025).

Demographic and clinical covariates were identified from the literature (Hu et al. 2014; Schulkes et al. 2018; Keating et al. 2021; Sachdev et al. 2024; Webber et al. 2024) and were those available in the data and included: age at death (categorized: 20–54, 55–64, 65–74, 75–84, ≥85), sex, Charlson comorbidity index (excluding malignancies) categorized as 0, 1, ≥2, cancer spread (localized, regional, metastatic), history of other malignancies (Y/N), mental health conditions (Y/N), tobacco use (Y/N), geographic location of residence (urban or rural), socioeconomic status, survival duration from diagnosis to death (i.e. 31–89, 90–179, ≥180 days), hospital type at last admission (public or private), and year of death.

## Results

A total of 9,476 colorectal cancer decedents were included. The median age at death was 74 years (IQR 13.4), and 54.4% were male. More than half of the cohort resided in urban areas, and around two-thirds had at least 1 comorbidity. In the last 30 days of life, 13.1% had >1 ED presentation, 9.2% had >1 hospital admission, 7.4% spent ≥14 days in hospital, 3.1% had ≥1 ICU admission, and 1.2% received ≥1 mechanical ventilation. In the last 90 days, 17.4% had ≥3 hospital admissions (Supplementary Table 1). A total of 1,488 (15.7%) people died in acute care. In the last 7 days of life, 3.1% received chemotherapy; in the last 14 days of life, 6.2% received chemotherapy; and 2.8% received radiotherapy in the last 30 days of life during 2016–2019 (Supplementary Table 2). Univariate and multivariable logistic regression examined factors associated with each indicator of potentially burdensome end-of-life care (Supplementary Tables 3–5).

Overall, 71.3% of people with colorectal cancer had no indicator of the composite indicator of potentially burdensome care, 18.9% had 1 indicator, and 9.8% had 2 or more indicators. The oldest group (≥85 years) of patients had the lowest (12.4%) proportion of ≥2 indicators of potentially burdensome end-of-life care. Men had a higher proportion of ≥2 indicators of potentially burdensome end-of-life care (61.1%) compared to women (38.9%). People living in urban areas had a higher proportion of 1 or ≥2 indicators of potentially burdensome end-of-life care than people living in rural locations. A higher proportion of patients whose last admission was to a private hospital received ≥1 indicator (20.4%) compared to no indicators (10.6%) of potentially burdensome end-of-life care. There was no difference in the proportion of people with none, 1, or 2 or more comorbidities by number of indicators of potentially burdensome end-of-life care (Table 2).

**Table 2.** Demographic characteristics of the cancer decedents by potentially burdensome end-of-life care composite index descriptive analysis Table 2 long description.

	**None** (*n* = 6,759; 71.3%)		**1 indicator** (*n* = 1,788; 18.9%)		**≥ 2 indicators** (*n* = 929; 9.8%)		
	n	%	n	%	n	%	*p*-Value
**Age at diagnosis**, median (IQR)	74	(13.4)	71	(13.7)	68	(13.8)	-
**Age group** (years)							
20–54	514	7.6	195	10.9	130	14.0	<0.0001
55–64	799	11.8	271	15.2	166	17.9	
65–74	1,486	22.0	466	26.1	271	29.2	
75–84	2,082	30.8	536	30.0	247	26.6	
≥85	1,878	27.8	320	17.9	115	12.4	
**Sex**							
Male	3,578	52.9	1,025	57.3	568	61.1	<0.0001
Female	3,181	47.1	763	42.7	361	38.9	
**Country of birth**							
Australia	4,644	68.7	1,320	73.8	696	74.9	<0.0001
Other/not known	2,115	31.3	468	26.2	233	25.1	
**Number of Charlson comorbidities, excluding malignancy**							
Nil	2,008	34.1	625	36.2	358	38.5	0.062
1 comorbidity	2,728	46.4	770	44.7	411	44.2	
≥2 comorbidities	1,148	19.5	329	19.1	160	17.2	
**Other comorbidities**							
Any mental health condition (yes)	780	13.3	190	11.0	85	9.1	<0.0001
Tobacco use (yes)	2,379	40.4	778	45.1	452	48.6	<0.0001
**Geographical location of residence^1^**							
Urban	4,037	69.1	1,063	62.3	568	62.1	<0.0001
Rural	1,805	30.9	644	37.7	346	37.9	
**Socioeconomic status**							
1 (most disadvantaged)	1,281	21.9	413	24.2	220	24.0	0.0002
2	1,461	25.0	498	29.2	256	27.9	
3	1,172	20.0	318	18.6	178	19.4	
4	803	13.7	188	11.0	101	11.0	
5 (least disadvantaged)	1,128	19.3	291	17.0	161	17.6	
**Year of death**							
2014	1,125	16.6	296	16.5	152	16.4	1.0
2015	1,147	17.0	307	17.2	156	16.8	
2016	1,134	16.8	302	16.9	160	17.2	
2017	1,136	16.8	295	16.5	155	16.7	
2018	1,107	16.4	308	17.2	158	17.0	
2019	1,110	16.4	280	15.7	148	15.9	
**Hospital type at last admission**							
Public	5,164	76.4	1,359	76.0	714	76.9	<0.0001
Private	720	10.6	365	20.4	^#^	^#^	
Not known	875	13.0	64	3.6	^#^	^#^	
**History of cancer** (yes)	1,619	24.7	464	26.5	248	27.5	0.09
**Survival duration** (days)							
31–89	457	7.0	146	8.3	68	7.5	0.003
≥90 days to <180	474	7.2	129	7.4	94	10.4	
≥180	5,622	85.8	1,478	84.3	741	82.1	
**Degree of cancer spread**							
In situ/localized	1,005	14.9	235	13.1	117	12.6	0.03
Regionalized	2,250	33.3	602	33.7	290	31.2	
Metastatic	2,567	38.0	725	40.6	396	42.6	
Not known	937	13.9	226	12.6	126	13.6	
**Place of death**^2^							
Home	1,146	17.0	154	8.6	41	4.4	<0.0001
Institutional care	4,173	61.7	1,295	72.4	700	75.4	
Not known	1,440	21.3	339	19.0	188	20.2	

# Cell sizes <5 or to prevent identification of cell sizes <5.

1 There were *n* = 2 not known geographical location of residence.

2 Institutional care includes: hospice, hospital, or residential aged care.

**Figure 1.**
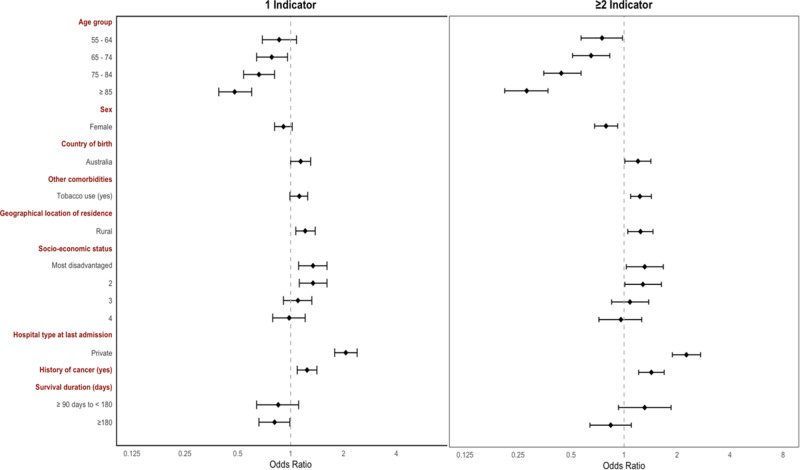
Multinomial model of characteristics associated with potentially burdensome care at the end-of-life for colorectal cancer patients^1^. Figure 1 long description.

^1^Reference categories for analysis were: nil composite indicators of potentially burdensome end-of-life care, age group 20–54, male, country of birth is other/unknown, nil comorbidities, urban, least disadvantaged, 2014 as year of death, public hospital type at last admission, survival duration of ≥180 days, in situ/localized cancer spread and place of death as home.

Multinomial regression showed a higher odds of ≥2 indicators of potentially burdensome end-of-life care among smokers (Adjusted Odds Ratio (AOR) 1.23, 95% CI 1.09–1.43), people with a history of prior cancer (AOR 1.43, 95% CI 1.21–1.69), who lived in a rural location (AOR 1.24, 95% CI 1.05–1.46), who lived in an area of low socioeconomic status (AOR 1.31, 95% CI 1.03–1.67), or who had a private hospital admission as their last admission (AOR 2.26, 95% CI 1.88–2.72) compared to no indicators of potentially burdensome end-of-life care and reference categories. Being older (AOR 0.28, 95% CI 0.21–0.37) and female (AOR 0.79, 95% CI 0.68–0.92) had a lower likelihood of receiving ≥2 indicators compared to no indicators and reference categories. Lower odds of receiving 1 indicator of potentially burdensome end-of-life care were found for people who had a longer survival duration (AOR 0.81, 95% CI 0.66–0.99) compared to no indicators (Figure 1 and Supplementary Table 7).

## Discussion

### Key results

Of 9,476 people who died from colorectal cancer in NSW, 15.7% died in acute care. An ED visit and >1 hospital admission were the most common indicators of potentially burdensome end-of-life care. A higher likelihood of experiencing ≥2 indicators of potentially burdensome end-of-life care was identified among smokers, patients with a prior history of cancer, people residing in rural areas, people living in socioeconomically disadvantaged areas, and people whose last admission was to a private hospital, whereas older age, female sex, and longer survival durations were associated with a lower likelihood.

### Findings in the context of other studies

#### Hospital service use

In the current study, 13.1% of patients had >1 ED presentation, and 9.2% had >1 hospital admission in the last 30 days of life (Table 3). In the Netherlands, Schulkes et al. (2018) (2011–2015) reported higher acute care use than the current study, with 55.9% of patients having >1 ED visit and 73.5% having >1 hospital admission in the last 90 days. The differences are likely due to the small sample size of colorectal cancer patients, consisting of 146 patients (24.2% of the total cohort), and the duration being 90 days, which reflects their conceptualization of the end-of-life period, compared to the 30-day timeframe in the current study (Schulkes et al. 2018).

**Table 3.** Comparison of potentially burdensome end-of-life care indicators for people who had a death from colorectal cancer by country and indicator type Table 3 long description.

Location	Timeframe	>1 ED visit^a^	>1 admission^a^	≥1 ICU stay^a^	Chemotherapy use^b^	Death in acute care
NSW, Australia (current study)	2014–2021	13.1	9.2	3.1	6.2	15.7
Alberta, Canada (Hu et al. 2014)	2006–2009	12.5	9.5	2.2	3.7	12.1
United States (Keating et al. 2021)	2011–2015	19.0	-	24.0	7.0	-
Ohio, US^c^ (Sachdev et al. 2024)	2012–2016	13.2	10.4	22.3	20.5	15.4
Netherlands^d^ (Schulkes et al. 2018)	2011–2015	55.9	73.5	-	46.6	32.4
Ontario, Canada^e^ (Webber et al. 2024)	2013–2018	-	-	10.0	28.4	-

a In the last 30 days of life.

b In the last 14 days of life.

c Chemotherapy use value is for “any cancer treatment.”

d All values were for the last 90 days of life.

e All values were for the last 100 days of life.

The present findings show 3.1% of people had ≥1 ICU admissions, whereas Keating et al. (2021) (United States, 2011–2015) found higher ICU use in the last 30 days of life (24%). This difference may reflect the United States healthcare system, where insurance structures allow more aggressive treatments than in Australia (Dzeng et al. 2018). Sachdev et al. (2024) (United States, 2012–2016) also showed high ICU use in the last 30 days of life (22.3%). These differences may be likely due to cohort selection (Table 3) (Sachdev et al. 2024). Webber et al. (2024) (Ontario, 2013–2018) reported that 10% had ≥1 ICU admissions in the last 100 days of life, which was a higher proportion than the current study that examined ≥1 ICU admissions in the last 30 days of life (4.7%) (Webber et al. 2024). This may reflect Canada’s healthcare structure prioritizing ICU admissions in the last 100 days of life, as well as the shorter timeframe in the present study (Chaudhuri et al. 2017).

#### Chemotherapy use

In the present analysis, 6.2% of patients reported chemotherapy use in the last 14 days of life. Hu et al. (2014) in Alberta, Canada (2006–2009) reported around half the chemotherapy use in the last 14 days of life at 3.7% compared to the current study. This difference is likely due to chemotherapy options expanding after 2010, influencing treatment decisions (Liu et al. 2024). Schulkes et al. (2018) reported 46.6% chemotherapy use in the last 90 days of life, and Webber et al. (2024) reported 28.4% chemotherapy use in the last 100 days before end-of-life, the difference likely due to the timeframe differences of 90 and 100 days versus 14 days for the current study.

### Characteristics associated with potentially burdensome indicators

#### Age group and sex

The current analysis found that older patients and women had a lower likelihood of receiving potentially burdensome end-of-life care. Similarly, Hu et al. 2014 observed that older patients (aged ≥61 years) were less likely to receive potentially burdensome end-of-life care than younger patients. This may reflect patient preferences to prioritize comfort-focused care, and clinicians may adopt less burdensome approaches due to frailty or perceived limited benefit (De Schreye et al. 2017). Webber et al. (2024) likewise reported that women experienced slightly lower rates than men, further supported by literature showing greater acceptance of palliative care by women (Gott et al. 2020).

#### All comorbidities and tobacco use

Consistent with present study findings, Sachdev et al. (2024) reported that a prior history of cancer increased the likelihood of potentially burdensome end-of-life care. Tobacco use was associated with higher odds of >1 ED presentations and a higher odds of ≥2 indicators of potentially burdensome end-of-life care in the current study, consistent with prior evidence (Just et al. 2021). Findings may indicate broader health and social disadvantage, including poorer baseline health due to tobacco-related comorbidities and delayed healthcare engagement (Just et al. 2021).

#### Residential location and socioeconomic status

The present study identified that living in a rural area was associated with a higher likelihood of >1 ED visit and ≥2 indicators of potentially burdensome end-of-life care. Similarly, Hu et al. (2014) reported that patients living in rural areas were more likely to experience >1 ED visit and >1 hospital admission in the last 30 days of life, reflecting limited availability or access to palliative care services, hence heavy reliance on ED and hospital services for symptom management. However, unlike current results, Webber et al. (2024) reported that higher-income patients had a higher likelihood of experiencing potentially burdensome end-of-life care compared to lower-income patients, which may reflect differences in access to, or barriers against, receipt of palliative or community-based care services in Ontario (Cai et al. 2017).

#### Private hospitals with the last admission

The current findings identified that people who had a death from colorectal cancer whose last admission occurred in a private hospital had higher odds for all individual indicators of potentially burdensome End-of-life-care (EOLC) except for >1 ED visit in the last 30 days of life and ≥2 indicators of potentially burdensome end-of-life care. This may be due to private hospitals acting as hospices or providing palliative care at the end-of-life, where these resources are not available in the community (Rosenwax et al. 2011). Private hospitals may have more resources for interventions and less integration with community palliative care, contributing to higher acute care use. Insurance incentives and patient or family expectations may also favor continued treatment near the end-of-life, even with limited benefit (Rosenwax et al. 2011).

### Impact on quality of life and the role of palliative care

The importance of palliative care is highlighted in the context of mitigating potentially burdensome end-of-life care. Palliative care referral has been reported to improve symptom control and reduce unnecessary hospitalizations (Hui et al. 2022). However, timely palliative care may be limited, specifically for people living in rural areas where geographic distance and workforce shortage remain barriers to access (Fasolino et al. 2024). These inequities may drive higher reliance on ED and acute care settings for end-of-life care (Cerni et al. 2023). It is important to note that lower health service use does not equate to better care, as it may reflect barriers to accessing hospital or palliative services, rather than patient or family preferences (Cerni et al. 2023).

It is important to acknowledge that health service use at the end-of-life may be necessary and is not unique to colorectal cancer (Grant et al. 2024). Some hospitalizations may be unavoidable, particularly for acute management of symptoms, such as pain or bleeding (Serra et al. 2023). To determine whether each care episode was potentially avoidable would have required expert clinical review of medical records, which was not possible for the current study.

## Strengths and limitations

The current study was a large population-based retrospective cohort. However, several limitations should be acknowledged. The reliance on administrative health data meant that patient preferences and quality of life could not be assessed. Future linkage with patient-reported data would be able to capture these aspects. The lack of patient-reported data limited the ability to evaluate whether the end- of-life care was truly “burdensome” from the patient’s perspective.

Chemotherapy and radiotherapy indicator data were only available between 2016 and 2019. Extending data to recent years would strengthen analyses. Furthermore, no data were available for service use, including primary care, community-based palliative, or home care services. There is likely under-representation of people with colorectal cancer who received palliative or hospice care and identification of aged care residents in hospital records.

As the study was limited to NSW, the generalizability of findings to other jurisdictions and countries is restricted. Comparative analyses across jurisdictions would clarify broader applicability. Data were not available on people who died from colorectal cancer in NSW, who lived near border towns and may have received treatment or hospital care in other jurisdictions, potentially impacting findings (Gabriel et al. 2015). Information on race and ethnicity was not available in the linked datasets, limiting the ability to examine potential disparities in end-of-life care across culturally diverse populations. Further studies may include developing predictive models to identify patients with colorectal cancer at greatest risk of receiving potentially burdensome end-of-life care.

## Conclusions

This study demonstrates that disparities exist in end-of-life care for people with colorectal cancer, particularly among socioeconomically disadvantaged and rural populations. By identifying where and for whom potentially burdensome end-of-life care occurs, this research highlights the need for policies and practices that promote high-quality, patient-centered end-of-life care; care that prioritizes dignity, comfort, and alignment with individual values.

## Supporting information

10.1017/S1478951526103095.sm001 Varghese et al. supplementary material Varghese et al. supplementary material

## Data Availability

The data that support the findings of this study are available from the NSW Ministry of Health and the NSW Cancer Institute. Restrictions apply to the availability of these data, which were used under license for the current study, so they are not publicly available.

## Supplementary material

The supplementary material for this article can be found at https://doi.org/10.1017/S1478951526103095.

## Table 1 Long description

The table lists indicators that population-based administrative health data can use to flag potentially burdensome care near the end of life. Utilization indicators include more than one emergency department visit in the last 30 days of life and more than one hospitalisation in the last 30 days, excluding palliative or hospice care. Intensive treatment indicators include at least one ICU admission in the last 30 days and mechanical ventilation in the last 30 days. Location and time-in-hospital indicators include death occurring in acute care (excluding palliative or hospice care) and spending more than 14 days in hospital during the last 30 days, again excluding palliative or hospice care. Cancer-treatment timing indicators include radiotherapy in the last 30 days and intravenous chemotherapy given very close to death, within the last 7 days or within the last 14 days. Each indicator is paired with citations showing it has been used in prior studies, but the table does not provide rates, counts, or thresholds beyond the time windows. Interpretation should consider that administrative records may not capture patient preferences, clinical appropriateness, or palliative intent consistently across settings.

Navigate back to Table 1.

## Table 2 Long description

The table compares demographic and clinical characteristics of cancer decedents across three groups: no potentially burdensome end-of-life care indicators (6,759; 71.3%), one indicator (1,788; 18.9%), and two or more indicators (929; 9.8%). Median age at diagnosis decreases as indicators increase (74, 71, and 68 years), and the share aged 85 or older drops from 27.8% with no indicators to 12.4% with two or more, while ages 20 to 54 rise from 7.6% to 14.0%. Men make up a larger proportion as indicators increase (52.9%, 57.3%, 61.1%), and tobacco use also rises (40.4%, 45.1%, 48.6%), while recorded mental health conditions decline (13.3% to 9.1%). Urban residence is more common in the no-indicator group (69.1%) than in the higher-indicator groups (about 62%), and disadvantage is slightly more concentrated in the higher-indicator groups (for example, the most disadvantaged category is about 24% versus 21.9%). Place of death shifts strongly away from home as indicators increase (17.0%, 8.6%, 4.4%) and toward institutional settings (61.7%, 72.4%, 75.4%). Metastatic disease is somewhat more common with more indicators (38.0% to 42.6%), and shorter survival (90 to under 180 days) is more frequent in the two-or-more group (10.4% versus 7.2%). Many differences are statistically significant, while Charlson comorbidity counts and history of cancer show little difference; some hospital-type cells are suppressed due to small counts.

Navigate back to Table 2.

## Figure 1 Long description

1 Indicator The x-axis is labeled Odds Ratio, ranging from 0.125 to 4, with tick labels at 0.125, 0.25, 0.5, 1, 2 and 4. A vertical dashed reference line is at 1. The y-axis lists: Age group with 55–64, 65–74, 75–84, 85 plus; Sex with Male and Female; Country of birth; Comorbidities; Other comorbidities; Indicator of cancer; Geographical location of residence; Socio-economic status; Not disadvantaged; 1; 2; 3; 4; Hospital type at last admission with Public and Private; History of cancer (yes); Survival duration (days) with 0 to 90 days and greater than 90 days to 1 year. Each row shows a point estimate with a horizontal confidence interval. The four age-group rows have point estimates left of 1. Male is at 1. Female is left of 1. Country of birth is near 1. Comorbidities is right of 1. Other comorbidities is near 1. Indicator of cancer is right of 1. Geographical location of residence is right of 1. Socio-economic status rows cluster around 1, with Not disadvantaged right of 1 and the numbered categories near 1. Public is near 1. Private is right of 1, around 2. History of cancer (yes) is right of 1, around 1.5. Survival duration (days) shows 0 to 90 days near 1 and greater than 90 days to 1 year left of 1. 2 Indicator The x-axis is labeled Odds Ratio, ranging from 0.125 to 8, with tick labels at 0.125, 0.25, 0.5, 1, 2, 4 and 8. A vertical dashed reference line is at 1. The y-axis lists the same categories as the left plot. Each row shows a point estimate with a horizontal confidence interval. The four age-group rows have point estimates left of 1. Male is at 1. Female is left of 1. Country of birth is near 1. Comorbidities is right of 1. Other comorbidities is near 1. Indicator of cancer is right of 1. Geographical location of residence is right of 1. Socio-economic status rows cluster around 1, with Not disadvantaged right of 1 and the numbered categories near 1. Public is near 1. Private is right of 1, around 2 to 3. History of cancer (yes) is right of 1, around 1.5 to 2. Survival duration (days) shows 0 to 90 days near 1 and greater than 90 days to 1 year left of 1.

Navigate back to Figure 1.

## Table 3 Long description

Percentages compare potentially burdensome end-of-life care indicators for people who died from colorectal cancer across regions and study periods. Measures include having more than one emergency department visit, more than one hospital admission, at least one intensive care stay, chemotherapy or other cancer treatment use, and death in acute care; some studies did not report every indicator. NSW, Australia (2014 to 2021) reports 13.1% with more than one ED visit, 9.2% with more than one admission, 3.1% with an ICU stay, 6.2% treatment use, and 15.7% dying in acute care. Alberta, Canada (2006 to 2009) is similar, with 12.5% more than one ED visit, 9.5% more than one admission, 2.2% ICU stay, 3.7% treatment use, and 12.1% death in acute care. The Netherlands (2011 to 2015) is much higher on reported measures: 55.9% more than one ED visit, 73.5% more than one admission, 46.6% treatment use, and 32.4% death in acute care. US studies show higher ICU and treatment use where reported: the United States overall (2011 to 2015) reports 19.0% more than one ED visit, 24.0% ICU stay, and 7.0% treatment use, while Ohio (2012 to 2016) reports 13.2% more than one ED visit, 10.4% more than one admission, 22.3% ICU stay, 20.5% treatment use, and 15.4% death in acute care. Ontario, Canada (2013 to 2018) reports 10.0% ICU stay and 28.4% treatment use, with other indicators not provided. Comparisons should be interpreted cautiously because the end-of-life window differs by indicator and study, and one study’s “chemotherapy” measure reflects any cancer treatment.

Navigate back to Table 3.
