# Supplementary material for: Potentially burdensome end-of-life care for colorectal cancer decedents: A retrospective cohort study
Source: Palliat Support Care. 2026 Jul 9;24:e190. doi: 10.1017/S1478951526103095 (PMC13370304; doi:10.1017/S1478951526103095)
Supplement: Varghese et al. supplementary material [file S1478951526103095sup001.docx]

**Supplementary Table 1: Demographic characteristics of the cancer decedents by potentially burdensome ED and hospital admission care indicators**

|  | **>1 ED visit in last 30 days**  (n=1241; 13.1%) | | **>1 admission in last 30 days**^1^ (n=874; 9.2%) | | **≥1 admission to ICU in last 30 days**  (n=297; 3.1%) | | **≥1 mechanical ventilation in last 30 days**  (n=113; 1.2%) | | **Place of death acute care**^1^ (n=1,488; 15.7%) | | **≥14 days in hospital in last 30 days**^1^  (n=705; 7.4%) | | **≥3 admissions in last 90 days**^1^  (n=1,651; 17.4%) | |
| --- | --- | --- | --- | --- | --- | --- | --- | --- | --- | --- | --- | --- | --- | --- |
|  | n | % | n | % | n | % | n | % | n | % | n | % | n | % |
| **Age group** (years) |  |  |  |  |  |  |  |  |  |  |  |  |  |  |
| 20–54 | 172 | 13.9 | 147 | 11.9 | 42 | 14.1 | 8 | 17.3 | 132 | 8.9 | 74 | 10.5 | 255 | 15.5 |
| 55–64 | 209 | 16.8 | 167 | 19.5 | 50 | 16.8 | 20 | 20.3 | 213 | 14.3 | 104 | 14.7 | 318 | 19.3 |
| 65–74 | 337 | 27.2 | 259 | 31.3 | 94 | 31.7 | 42 | 35.4 | 400 | 26.9 | 198 | 28.1 | 520 | 31.5 |
| 75–84 | 340 | 27.4 | 213 | 26.7 | 75 | 25.3 | 28 | 21.8 | 470 | 31.6 | 205 | 29.1 | 412 | 24.9 |
| ≥85 | 183 | 14.7 | 88 | 10.6 | 36 | 12.1 | 15 | 5.2 | 273 | 18.3 | 124 | 17.6 | 146 | 8.8 |
| **Sex** |  |  |  |  |  |  |  |  |  |  |  |  |  |  |
| Male | 750 | 60.4 | 522 | 60.4 | 190 | 64.0 | 72 | 63.7 | 853 | 57.3 | 405 | 57.5 | 977 | 59.2 |
| Female | 491 | 39.6 | 352 | 39.6 | 107 | 36.0 | 41 | 36.3 | 635 | 42.7 | 300 | 42.5 | 674 | 40.8 |
| **Country of birth** |  |  |  |  |  |  |  |  |  |  |  |  |  |  |
| Australia | 939 | 75.7 | 657 | 75.2 | 203 | 68.4 | 78 | 69.0 | 1113 | 74.8 | 507 | 71.9 | 1208 | 73.2 |
| Other/not known | 302 | 24.3 | 217 | 24.8 | 94 | 31.6 | 35 | 31.0 | 375 | 25.2 | 198 | 28.1 | 443 | 26.8 |
| **Number of Charlson comorbidities, excluding malignancy** | | | |  |  |  |  |  |  |  |  |  |  |  |
| Nil | 416 | 33.5 | 337 | 38.6 | 124 | 41.8 | 55 | 48.7 | 575 | 38.6 | 278 | 39.4 | 703 | 42.6 |
| 1 comorbidity | 545 | 43.9 | 404 | 46.2 | 107 | 36.0 | 33 | 29.2 | 641 | 43.1 | 289 | 41.0 | 736 | 44.6 |
| ≥2 comorbidities | 216 | 17.4 | 133 | 15.2 | 66 | 22.2 | 25 | 22.1 | 272 | 18.3 | 138 | 19.6 | 212 | 12.8 |
| **Other comorbidities** |  |  |  |  |  |  |  |  |  |  |  |  |  |  |
| Any mental health condition (yes) | 126 | 10.7 | 66 | 7.6 | 28 | 9.4 | 12 | 10.6 | 157 | 10.5 | 87 | 12.3 | 110 | 6.7 |
| Tobacco use (yes) | 592 | 50.3 | 408 | 46.7 | 144 | 48.5 | 59 | 52.2 | 676 | 45.4 | 335 | 47.5 | 790 | 47.8 |
| **Geographical location of residence^2^** | | | | | | | | | | | | | | |
| Urban | 615 | 49.5 | 564 | 65.5 | 220 | 74.1 | 75 | 66.4 | 949 | 64.8 | 481 | 68.2 | 1,080 | 66.4 |
| Rural | 552 | 44.5 | 295 | 34.3 | 75 | 25.2 | 36 | 31.8 | 515 | 35.1 | 216 | 30.6 | 545 | 33.5 |
| **Socio-economic status^3^** |  |  |  |  |  |  |  |  |  |  |  |  |  |  |
| Most disadvantaged | 345 | 27.8 | 172 | 19.7 | 69 | 23.2 | 29 | 25.7 | 327 | 22.0 | 155 | 22.0 | 305 | 18.5 |
| 2 | 380 | 30.6 | 232 | 26.5 | 67 | 22.6 | 21 | 18.6 | 399 | 26.8 | 176 | 25.0 | 394 | 23.9 |
| 3 | 208 | 16.8 | 171 | 19.6 | 58 | 19.5 | 22 | 19.5 | 287 | 19.3 | 133 | 18.9 | 334 | 20.2 |
| 4 | 121 | 9.7 | 97 | 11.1 | 36 | 12.1 | 12 | 10.6 | 166 | 11.2 | 76 | 10.8 | 212 | 12.8 |
| Least disadvantaged | 114 | 9.2 | 188 | 21.5 | 65 | 21.9 | 27 | 23.9 | 287 | 19.3 | 157 | 22.3 | 382 | 23.1 |
| **Year of death** |  |  |  |  |  |  |  |  |  |  |  |  |  |  |
| 2014 | 192 | 15.5 | 144 | 16.5 | 43 | 14.1 | 20 | 17.7 | 267 | 17.9 | 132 | 18.7 | 262 | 15.9 |
| 2015 | 206 | 16.6 | 154 | 17.6 | 47 | 15.8 | 15 | 13.3 | 254 | 17.1 | 130 | 18.4 | 262 | 15.9 |
| 2016 | 205 | 16.5 | 146 | 16.7 | 51 | 17.2 | 17 | 15.0 | 267 | 17.9 | 123 | 17.4 | 287 | 17.4 |
| 2017 | 201 | 16.2 | 133 | 15.2 | 51 | 17.2 | 20 | 17.7 | 257 | 17.3 | 109 | 15.5 | 286 | 17.3 |
| 2018 | 227 | 18.3 | 154 | 17.6 | 52 | 17.5 | 22 | 19.5 | 237 | 15.9 | 114 | 16.2 | 278 | 16.8 |
| 2019 | 210 | 16.9 | 143 | 16.4 | 54 | 18.2 | 19 | 16.8 | 206 | 13.8 | 97 | 13.8 | 276 | 16.7 |
| **Hospital type at last admission^4^** |  |  |  |  |  |  |  |  |  |  |  |  |  |  |
| Public | 1126 | 90.7 | 636 | 72.8 | 232 | 78.1 | 92 | 81.4 | 1001 | 67.3 | 480 | 68.1 | 1156 | 70.0 |
| Private | 51 | 4.1 | 238 | 27.2 | 65 | 21.9 | 21 | 18.6 | 487 | 32.7 | 225 | 31.9 | 495 | 30.0 |
| **Age at diagnosis,** median (IQR) | 69.0 | (19.0) | 66.0 | (19.0) | 69.0 | (17.0) | 71.0 | (16.0) | 72.0 | (17.0) | 71.0 | (18.0) | 66.0 | (18.0) |
| **Time from diagnosis to death** (years), median (IQR) | 2.2 | (4.4) | 2.1 | (3.9) | 1.7 | (5.0) | 1.3 | (4.8) | 2.2 | (4.9) | 2.3 | (4.7) | 2.1 | (3.8) |
| **History of cancer** (yes) | 304 | 25.0 | 219 | 25.9 | 88 | 30.2 | 39 | 35.8 | 424 | 29.2 | 203 | 29.4 | 387 | 24.0 |
| **Survival duration** (days)^5^ |  |  |  |  |  |  |  |  |  |  |  |  |  |  |
| 31–89 | 75 | 6.0 | 47 | 5.4 | 46 | 15.5 | 23 | 20.9 | 137 | 9.2 | 65 | 9.2 | 147 | 8.9 |
| ≥90 days to <180 | 99 | 8.0 | 74 | 8.5 | 42 | 14.1 | 16 | 14.5 | 124 | 8.3 | 54 | 7.7 | 115 | 7.0 |
| ≥180 | 1041 | 83.9 | 725 | 82.9 | 203 | 68.4 | 71 | 64.5 | 1193 | 80.2 | 572 | 81.1 | 1350 | 81.8 |
| **Degree of cancer spread** |  |  |  |  |  |  |  |  |  |  |  |  |  |  |
| In-situ/localised | 160 | 12.9 | 95 | 10.9 | 46 | 15.5 | 24 | 21.2 | 199 | 13.4 | 98 | 13.9 | 182 | 11.0 |
| Regionalised | 397 | 32.0 | 253 | 29.0 | 107 | 36.0 | 43 | 38.1 | 509 | 34.2 | 263 | 37.3 | 502 | 30.4 |
| Metastatic | 511 | 41.2 | 407 | 46.6 | 108 | 36.4 | 31 | 27.4 | 589 | 39.6 | 264 | 37.5 | 787 | 47.7 |
| Not known | 173 | 13.9 | 119 | 13.6 | 36 | 12.1 | 15 | 13.3 | 191 | 12.8 | 80 | 11.3 | 180 | 10.9 |
| **Place of death^6^** |  |  |  |  |  |  |  |  |  |  |  |  |  |  |
| Home | 137 | 11.0 | 78 | 8.9 | 16 | 5.4 | # | # | 5 | 0.3 | 36 | 5.1 | 195 | 11.8 |
| Institutional care | 851 | 68.6 | 617 | 70.6 | 217 | 73.1 | 89 | 78.8 | 1219 | 81.9 | 553 | 78.4 | 1127 | 68.3 |
| Not known | 173 | 13.9 | 119 | 13.6 | 36 | 12.1 | # | # | 191 | 12.8 | 80 | 11.3 | 180 | 10.9 |
| ^1^ Excluding palliative or hospice care. # Cell sizes <5 or to prevent identification of cell sizes <5. ^2^ There were n=3 not known geographic location of residence. ^3^ There were n=145 not known socio-economic status. ^4^ There were n=64 not known hospital type at last admission. ^5^ There were n=150 not known survival duration. ^6^ Institutional care includes: hospice, hospital or residential aged care. | | | | | | | | | | | | | | |

**Supplementary Table 2: Demographic characteristics of the cancer decedents by potentially burdensome chemotherapy or radiotherapy indicators during 2016–2019**

|  | **Last dose chemotherapy in last 7 days**  (n=194; 3.1%) | | **Last dose chemotherapy in last 14 days**  (n=388; 6.2%) | | **Radiotherapy in last 30 days**  (n=177; 2.8%) | |
| --- | --- | --- | --- | --- | --- | --- |
|  | n | % | n | % | n | % |
| **Age group** (years) |  |  |  |  |  |  |
| 20–54 | 53 | 27.3 | 81 | 20.9 | 30 | 16.9 |
| 55–64 | 39 | 20.1 | 79 | 20.3 | 43 | 24.3 |
| 65–74 | 49 | 25.3 | 119 | 30.7 | 47 | 26.6 |
| ≥75 | 53 | 27.3 | 109 | 28.1 | 57 | 32.2 |
| **Sex** |  |  |  |  |  |  |
| Male | 120 | 61.9 | 245 | 63.1 | 104 | 58.8 |
| Female | 74 | 38.1 | 143 | 36.9 | 73 | 41.2 |
| **Country of birth** |  |  |  |  |  |  |
| Australia | 129 | 65.0 | 268 | 69.1 | 115 | 65.0 |
| Other/not known | 68 | 35.1 | 120 | 30.9 | 62 | 35.0 |
| **Number of Charlson comorbidities, excluding malignancy^1^** |  |  |  |  |  |  |
| Nil | 75 | 39.1 | 146 | 38.2 | 63 | 35.8 |
| 1 comorbidity | 86 | 44.8 | 165 | 43.2 | 91 | 51.7 |
| ≥2 comorbidities | 31 | 16.2 | 71 | 18.6 | 22 | 12.5 |
| **Other comorbidities** |  |  |  |  |  |  |
| Any mental health condition (yes) | 12 | 6.3 | 26 | 6.8 | 24 | 13.6 |
| Tobacco use (yes) | 93 | 48.4 | 173 | 45.3 | 86 | 48.9 |
| **Geographical location of residence^2^** |  |  |  |  |  |  |
| Urban | 134 | 69.8 | 260 | 68.6 | 131 | 74.4 |
| Rural | 57 | 29.7 | 118 | 31.1 | 45 | 25.6 |
| **Socio-economic status^3^** |  |  |  |  |  |  |
| Most disadvantaged | 56 | 29.2 | 99 | 26.1 | 41 | 23.3 |
| 2 | 46 | 24.0 | 100 | 26.4 | 33 | 18.7 |
| 3 | 39 | 20.3 | 75 | 19.8 | 35 | 19.9 |
| 4 | 29 | 15.1 | 49 | 12.9 | 32 | 18.2 |
| Least disadvantaged | 22 | 11.4 | 56 | 14.8 | 35 | 19.9 |
| **Year of death** |  |  |  |  |  |  |
| 2016 | 51 | 26.3 | 99 | 25.5 | 49 | 27.7 |
| 2017 | 55 | 28.4 | 103 | 26.5 | 40 | 22.6 |
| 2018 | 48 | 24.7 | 102 | 26.3 | 41 | 23.2 |
| 2019 | 40 | 20.6 | 84 | 21.7 | 47 | 26.5 |
| **Hospital type at last admission^4^** |  |  |  |  |  |  |
| Public | 162 | 83.5 | 321 | 82.7 | 154 | 87.0 |
| Private | 30 | 15.5 | 61 | 15.7 | 22 | 12.4 |
| **Age at diagnosis**^5^**,** median (SD) | 64.0 | (14.7) | 65.0 | (13.7) | 65.0 | (14.6) |
| **Time from diagnosis to death**^5^  (years), median (SD) | 1.9 | (4.7) | 2.1 | (4.7) | 2.1 | (5.2) |
| **History of cancer** (yes) | 37 | 19.1 | 76 | 19.8 | 41 | 23.6 |
| **Survival duration** (days)^6^ |  |  |  |  |  |  |
| 31–89 | 18 | 9.3 | 31 | 8.1 | 20 | 11.5 |
| ≥90 days to <180 | 16 | 8.2 | 34 | 8.9 | 16 | 9.2 |
| ≥180 | 160 | 82.5 | 319 | 83.1 | 138 | 79.3 |
| **Degree of cancer spread^7^** |  |  |  |  |  |  |
| In-situ/localised | 16 | 8.2 | 29 | 7.5 | 22 | 12.4 |
| Regionalised | 47 | 24.2 | 117 | 30.2 | 56 | 31.6 |
| Metastatic | 113 | 58.2 | 207 | 53.4 | 81 | 45.8 |
| Not known | 18 | 9.3 | 35 | 9.0 | 18 | 10.2 |
| **Place of death^8,9^** |  |  |  |  |  |  |
| Home | 23 | 11.9 | 42 | 10.8 | 17 | 9.6 |
| Institutional care | 128 | 66.0 | 249 | 64.2 | 108 | 61.0 |
| Not known | 43 | 22.2 | 97 | 25.0 | 52 | 29.4 |

^1^ There were n=9 not known number of Charlson comorbidities. ^2^ There were n=3 not known geographical location of residence. ^3^ There were n=12 not known socio-economic status. ^4^ There were n=9 not known hospital type at last admission. ^5^ Diagnosis age and time from diagnosis to death missing for 28, 76 and 50 decedents for last dose chemotherapy in 7 days, in 14 days and radiotherapy in last 30 days, respectively. ^6^ There were n=7 not known survival duration. ^7^ There were n=71 not known degree of cancer spread. ^8^ There were n=192 not known place of death. ^9^ Institutional care includes: hospice, hospital or residential aged care.

**Supplementary Table 3: Univariate analysis of factors associated with potentially burdensome end-of-life care indicators among cancer decedents**

|  | **>1 ED visit in last 30 days** | | **>1 admission in last 30 days** (n=874; 13.7%) | | **≥1 admission to ICU in last 30 days**  (n=297; 4.7%) | | **Place of death acute care** (n=1,488; 23.4%) | | **≥14 days in hospital in last 30 days**  (n=705; 11.1%) | | **≥3 admissions in last 90 days**  (n=1,651; 25.9%) | |
| --- | --- | --- | --- | --- | --- | --- | --- | --- | --- | --- | --- | --- |
|  | OR | 95% CI | OR | 95% CI | OR | 95% CI | OR | 95% CI | OR | 95% CI | OR | 95% CI |
| **Age group** (years) |  |  |  |  |  |  |  |  |  |  |  |  |
| 20–54 | 1 |  | 1 |  | 1 |  | 1 |  | 1 |  | 1 |  |
| 55–64 | 0.79 | 0.63-0.99 | 0.74* | 0.58-0.94 | 0.80 | 0.53-1.22 | 1.12 | 0.88-1.41 | 0.95 | 0.70-1.30 | 0.79* | 0.65-0.96 |
| 65–74 | 0.69*** | 0.57-0.85 | 0.62*** | 0.50-0.77 | 0.84 | 0.58-1.22 | 1.18 | 0.95-1.46 | 1.01 | 0.76-1.34 | 0.70*** | 0.59-0.83 |
| 75–84 | 0.52*** | 0.43-0.64 | 0.38*** | 0.30-0.47 | 0.51** | 0.35-0.75 | 1.05 | 0.85-1.30 | 0.80 | 0.60-1.05 | 0.38*** | 0.32-0.46 |
| ≥85 | 0.33*** | 0.27-0.42 | 0.19*** | 0.14-0.25 | 0.30*** | 0.19-0.47 | 0.73** | 0.57-0.90 | 0.59*** | 0.43-0.79 | 0.15*** | 0.12-0.19 |
| **Sex** |  |  |  |  |  |  |  |  |  |  |  |  |
| Male | 1 |  | 1 |  | 1 |  | 1 |  | 1 |  | 1 |  |
| Female | 0.76*** | 0.67-0.86 | 0.79** | 0.69-0.91 | 0.67** | 0.53-0.85 | 0.88* | 0.78-0.98 | 0.88 | 0.75-1.03 | 0.80*** | 0.72-0.89 |
| **Country of birth** |  |  |  |  |  |  |  |  |  |  |  |  |
| Australia | 1.37*** | 1.19-1.57 | 1.31** | 1.12-1.54 | 0.91 | 0.71-1.17 | 1.31*** | 1.15-1.48 | 1.09 | 0.92-1.29 | 1.19** | 1.05-1.34 |
| Other/not known | 1 |  | 1 |  | 1 |  | 1 |  | 1 |  | 1 |  |
| **Number of Charlson comorbidities, excluding malignancy** | | | |  |  |  |  |  |  |  |  |  |
| Nil | 1 |  | 1 |  | 1 |  | 1 |  | 1 |  | 1 |  |
| 1 comorbidity | 1.00 | 0.87-1.15 | 0.91 | 0.78-1.06 | 0.65** | 0.50-0.85 | 0.82** | 0.73-0.93 | 0.78** | 0.64-0.93 | 0.75*** | 0.67-0.85 |
| ≥2 comorbidities | 0.94 | 0.79-1.12 | 0.70** | 0.56-0.86 | 0.97 | 0.72-1.32 | 0.84* | 0.71-0.98 | 0.90 | 0.73-1.11 | 0.48*** | 0.41-0.57 |
| **Other comorbidities** |  |  |  |  |  |  |  |  |  |  |  |  |
| Any mental health condition (yes) | 0.83 | 0.68-1.01 | 0.55*** | 0.43-0.71 | 0.73 | 0.49-1.08 | 0.81* | 0.68-0.97 | 1.00 | 0.79-1.26 | 0.45*** | 0.37-0.55 |
| Tobacco use (yes) | 1.46*** | 1.29-1.65 | 1.22** | 1.06-1.40 | 1.30 | 1.03-1.64 | 1.17** | 1.04-1.30 | 1.26** | 1.08-1.47 | 1.32*** | 1.19-1.47 |
| **Geographical location of residence^1^** |  |  |  |  |  |  |  |  |  |  |  |  |
| Urban | 1 |  | 1 |  | 1 |  | 1 |  | 1 |  | 1 |  |
| Rural | 2.02*** | 1.78-2.29 | 1.10 | 0.92-1.24 | 0.68** | 0.52-0.89 | 1.12 | 1.00-1.26 | 0.90 | 0.76-1.07 | 1.03 | 0.92-1.15 |
| **Socio-economic status^2^** |  |  |  |  |  |  |  |  |  |  |  |  |
| Most disadvantaged | 2.83*** | 2.26-3.54 | 0.73** | 0.59-0.91 | 0.87 | 0.62-1.23 | 0.93 | 0.78-1.11 | 0.80 | 0.63-1.01 | 0.59*** | 0.50-0.70 |
| 2 | 2.66*** | 2.14-3.32 | 0.87 | 0.71-1.06 | 0.73 | 0.51-1.03 | 0.99 | 0.84-1.17 | 0.78 | 0.62-0.98 | 0.68*** | 0.58-0.80 |
| 3 | 1.83*** | 1.44-2.33 | 0.85 | 0.68-1.05 | 0.84 | 0.59-1.20 | 0.94 | 0.78-1.12 | 0.79* | 0.62-1.00 | 0.79** | 0.66-0.93 |
| 4 | 1.60** | 1.23-2.10 | 0.72 | 0.56-0.93 | 0.79 | 0.52-1.20 | 0.81* | 0.66-1.00 | 0.68** | 0.51-0.90 | 0.76** | 0.63-0.91 |
| Least disadvantaged | 1 |  | 1 |  | 1 |  | 1 |  | 1 |  | 1 |  |
| **Year of death** |  |  |  |  |  |  |  |  |  |  |  |  |
| 2014 | 1 |  | 1 |  | 1 |  | 1 |  | 1 |  | 1 |  |
| 2015 | 1.06 | 0.86-1.30 | 1.05 | 0.83-1.33 | 1.10 | 0.72-1.67 | 0.92 | 0.76-1.11 | 0.96 | 0.74-1.23 | 0.97 | 0.81-1.17 |
| 2016 | 1.06 | 0.86-1.31 | 1.0 | 0.78-1.27 | 1.20 | 0.80-1.82 | 0.98 | 0.82-1.18 | 0.91 | 0.71-1.18 | 1.10 | 0.91-1.32 |
| 2017 | 1.04 | 0.84-1.29 | 0.91 | 0.71-1.16 | 1.21 | 0.80-1.83 | 0.95 | 0.78-1.14 | 0.81 | 0.62-1.05 | 1.10 | 0.92-1.32 |
| 2018 | 1.21 | 0.99-1.49 | 1.08 | 0.85-1.37 | 1.25 | 0.82-1.89 | 0.87 | 0.72-1.05 | 0.85 | 0.66-1.11 | 1.07 | 0.89-1.29 |
| 2019 | 1.14 | 0.92-1.40 | 1.02 | 0.80-1.30 | 1.33 | 0.88-2.00 | 0.76** | 0.62-0.92 | 0.73* | 0.56-0.96 | 1.09 | 0.91-1.32 |
| **Hospital type at last admission^3^** |  |  |  |  |  |  |  |  |  |  |  |  |
| Public | 1 |  | 1 |  | 1 |  | 1 |  | 1 |  | 1 |  |
| Private | 0.22*** | 0.17-0.30 | 2.33*** | 1.98-2.74 | 1.59** | 1.20-2.11 | 3.73*** | 3.27-4.25 | 2.95*** | 2.48-3.50 | 3.23*** | 2.84-2.68 |
| **History of cancer** (yes) | 0.98 | 0.85-1.13 | 1.03 | 0.88-1.22 | 1.29* | 1.00-1.66 | 1.26*** | 1.11-1.43 | 1.25* | 1.05-1.48 | 0.92 | 0.81-1.04 |
| **Survival duration** (days)^4^ |  |  |  |  |  |  |  |  |  |  |  |  |
| 31–89 | 0.82 | 0.64-1.05 | 0.74 | 0.54-1.00 | 2.77*** | 1.99-3.85 | 1.43*** | 1.17-1.74 | 1.36* | 1.04-1.78 | 1.35** | 1.11-1.63 |
| ≥90 days to <180 | 1.08 | 0.87-1.35 | 1.17 | 0.91-1.50 | 2.42*** | 1.71-3.40 | 1.21 | 0.98-1.48 | 1.07 | 0.80-1.43 | 0.95 | 0.77-1.17 |
| ≥180 | 1 |  | 1 |  | 1 |  | 1 |  | 1 |  | 1 |  |
| **Degree of cancer spread** |  |  |  |  |  |  |  |  |  |  |  |  |
| In-situ/localised | 1 |  | 1 |  | 1 |  | 1 |  | 1 |  | 1 |  |
| Regionalised | 1.08 | 0.89-1.32 | 1.16 | 0.91-1.49 | 1.00 | 0.71-1.43 | 1.12 | 0.94-1.34 | 1.17 | 0.92-1.49 | 1.23* | 1.02-1.47 |
| Metastatic | 1.20 | 1.00-1.45 | 1.65*** | 1.31-2.08 | 0.86 | 0.61-1.22 | 1.11 | 0.92-1.32 | 0.99 | 0.78-1.26 | 1.75*** | 1.47-2.09 |
| Not known | 1.16 | 0.92-1.46 | 1.35* | 1.02-1.79 | 0.82 | 0.53-1.28 | 1.01 | 0.82-1.26 | 0.85 | 0.63-1.15 | 1.05 | 0.84-1.31 |
| *p* < 0.001 (***), *p* < 0.01 (**), *p* ≤ 0.05 (*); OR: Unadjusted Odds Ratios. ^1^ There were n=3 not known geographic location of residence. ^2^ There were n=145 not known socio-economic status. ^3^ There were n=64 not known hospital type at last admission. ^4^ There were n=150 not known survival duration. ^5^Mechanical ventilation was not analysed using logistic regression due to low sample size. | | | | | | | | | | | | |

**Supplementary Table 4: Univariate analysis of factors associated with potentially burdensome chemotherapy or radiotherapy indicators during 2016-2019**

|  | **Last dose chemotherapy in last 7 days**  (n=194; 25.6%) | | **Last dose chemotherapy in last 14 days**  (n=388; 51.1%) | | **Radiotherapy in last 30 days**  (n=177; 23.3%) | |
| --- | --- | --- | --- | --- | --- | --- |
|  | OR | 95% CI | OR | 95% CI | OR | 95% CI |
| **Age group** |  |  |  |  |  |  |
| 20–54 | 1 |  | 1 |  | 1 |  |
| 55–64 | 0.47** | 0.30-0.72 | 0.62** | 0.44-0.86 | 0.97 | 0.60-1.58 |
| 65–74 | 0.33*** | 0.22-0.50 | 0.51*** | 0.37-0.69 | 0.60* | 0.37-0.96 |
| ≥75 | 0.16*** | 0.11-0.23 | 0.20*** | 0.15-0.27 | 0.34*** | 0.21-0.53 |
| **Sex** |  |  |  |  |  |  |
| Male | 1 |  | 1 |  | 1 |  |
| Female | 0.76 | 0.57-1.03 | 0.71** | 0.57-0.88 | 0.86 | 0.64-1.18 |
| **Country of birth** |  |  |  |  |  |  |
| Australia | 0.76* | 0.56-1.03 | 0.92 | 0.74-1.15 | 0.76 | 0.56-1.04 |
| Other/not known | 1 |  | 1 |  | 1 |  |
| **Number of Charlson comorbidities, excluding malignancy^1^** | | | |  |  |  |
| Nil | 1 |  | 1 |  | 1 |  |
| 1 comorbidity | 0.86 | 0.63-1.18 | 0.86 | 0.68-1.09 | 1.13 | 0.81-1.58 |
| ≥2 comorbidities | 0.75 | 0.49-1.15 | 0.91 | 0.68-1.22 | 0.66 | 0.40-1.08 |
| **Other comorbidities** |  |  |  |  |  |  |
| Any mental health condition (yes) | 0.45** | 0.25-0.81 | 0.49*** | 0.32-0.73 | 1.10 | 0.71-1.70 |
| Tobacco use (yes) | 1.30 | 0.97-1.73 | 1.15 | 0.93-1.41 | 1.38* | 1.02-1.87 |
| **Geographical location of residence^2^** |  |  |  |  |  |  |
| Urban | 1 |  | 1 |  | 1 |  |
| Rural | 0.86 | 0.63-1.17 | 0.92 | 0.73-1.15 | 0.68* | 0.48-0.97 |
| **Socio-economic status^3^** |  |  |  |  |  |  |
| Most disadvantaged | 2.31** | 1.39-3.84 | 1.60** | 1.13-2.25 | 1.05 | 0.66-1.67 |
| 2 | 1.57 | 0.93-2.64 | 1.33 | 0.95-1.87 | 0.68 | 0.41-1.11 |
| 3 | 1.81* | 1.06-3.09 | 1.36 | 0.95-1.94 | 1.02 | 0.63-1.65 |
| 4 | 2.06* | 1.17-3.64 | 1.35 | 0.91-2.01 | 1.44 | 0.87-2.36 |
| Least disadvantaged | 1 |  | 1 |  | 1 |  |
| **Year of death** |  |  |  |  |  |  |
| 2016 | 1 |  | 1 |  | 1 |  |
| 2017 | 1.09 | 0.74-1.61 | 1.06 | 0.79-1.41 | 0.82 | 0.53-1.26 |
| 2018 | 0.91 | 0.60-1.36 | 1.01 | 0.75-1.36 | 0.84 | 0.55-1.29 |
| 2019 | 0.82 | 0.54-1.25 | 0.90 | 0.67-1.22 | 1.03 | 0.68-1.55 |
| **Hospital type at last admission^4^** |  |  |  |  |  |  |
| Public | 1 |  | 1 |  | 1 |  |
| Private | 0.96 | 0.65-1.44 | 0.98 | 0.73-1.31 | 0.70 | 0.44-1.12 |
| **History of cancer** (yes) | 0.70 | 0.49-1.01 | 0.73* | 0.56-0.95 | 0.91 | 0.64-1.30 |
| **Survival duration** (days)^5^ |  |  |  |  |  |  |
| 31–89 | 1 |  | 1 |  | 1 |  |
| ≥90 days to <180 | 0.85 | 0.43-1.69 | 1.06 | 0.64-1.75 | 0.80 | 0.41-1.58 |
| ≥180 | 0.80 | 0.49-1.31 | 0.92 | 0.63-1.35 | 0.66 | 1.08 |
| **Degree of cancer spread^6^** |  |  |  |  |  |  |
| In-situ/localised | 1 |  | 1 |  | 1 |  |
| Regionalised | 1.22 | 0.68-2.20 | 1.67* | 1.09-2.54 | 1.01 | 0.61-1.66 |
| Metastatic | 2.51** | 1.45-4.32 | 2.48*** | 1.66-3.72 | 1.18 | 0.73-1.91 |
| Not known | 1.43 | 0.71-2.88 | 1.36 | 0.80-2.30 | 0.85 | 0.44-1.65 |
| *p* < 0.001 (***), *p* < 0.01 (**), *p* ≤ 0.05 (*); OR: Unadjusted Odds Ratios.^1^ There were n=9 not known number of Charlson comorbidities. ^2^ There were n=3 not known geographical location of residence. ^3^ There were n=12 not known socio-economic status. ^4^ There were n=9 not known hospital type at last admission. ^5^There were n=7 not known survival duration. ^6^ There were n=71 not known degree of cancer spread. | | | | | | |

**Supplementary Table 5: Multivariable analysis of factors associated with potentially burdensome end-of-life care indicators among cancer decedents**

|  | **>1 ED visit in last 30 days** | | **>1 admission in last 30 days** (n=874; 13.7%) | | **≥1 admission to ICU in last 30 days**  (n=297; 4.7%) | | **Place of death acute care** (n=1,488; 23.4%) | | **≥14 days in hospital in last 30 days**  (n=705; 11.1%) | | **≥3 admissions in last 90 days**  (n=1,651; 25.9%) | |
| --- | --- | --- | --- | --- | --- | --- | --- | --- | --- | --- | --- | --- |
|  | AOR | 95% CI | AOR | 95% CI | AOR | 95% CI | AOR | 95% CI | AOR | 95% CI | AOR | 95% CI |
| **Age group** |  |  |  |  |  |  |  |  |  |  |  |  |
| 20-54 | 1 |  | 1 |  | 1 |  | 1 |  | 1 |  | 1 |  |
| 55-64 | 0.73** | 0.58-0.92 | 0.72* | 0.56-0.93 | 0.69 | 0.45-1.07 | 1.01 | 0.79-1.30 | 0.85 | 0.61-1.17 | 1.73** | 0.60-0.90 |
| 65-74 | 0.62*** | 0.50-0.77 | 0.61*** | 0.49-0.77 | 0.71 | 0.48-1.05 | 1.09 | 0.87-1.36 | 0.90 | 0.67-1.20 | 1.68*** | 0.56-0.82 |
| 75-84 | 0.46*** | 0.37-0.57 | 0.39*** | 0.31-0.49 | 0.38*** | 0.26-0.58 | 1.02 | 0.82-1.28 | 0.73** | 0.54-0.98 | 0.39*** | 0.32-0.48 |
| ≥85 | 0.34*** | 0.26-0.43 | 0.22*** | 0.17-0.30 | 0.23*** | 0.14-0.37 | 0.79 | 0.62-1.00 | 0.59*** | 0.43-0.81 | 0.18*** | 0.14-0.23 |
| **Sex** |  |  |  |  |  |  |  |  |  |  |  |  |
| Male | 1 |  | 1 |  | 1 |  | - |  | - |  | 1 |  |
| Female | 0.86* | 0.75-0.98 | 0.84* | 0.73-0.98 | 0.70** | 0.55-0.90 | - | - | - | - | 0.87* | 0.78-0.98 |
| **Country of birth** |  |  |  |  |  |  |  |  |  |  |  |  |
| Australia | 1.18* | 1.01-1.38 | 1.24* | 1.05-1.47 | - | - | - | - | - | - | - | - |
| Other/not known | 1 |  | 1 |  | - |  | - |  | - |  | - |  |
| **Number of Charlson comorbidities, excluding malignancy^1^** | | | |  |  |  |  |  |  |  |  |  |
| Nil | - |  | - |  | 1 |  | 1 |  | 1 |  | 1 |  |
| 1 comorbidity | - | - | - | - | 0.65** | 0.50-0.86 | 0.85* | 0.75-0.97 | 0.81* | 0.68-0.97 | 0.72*** | 0.63-0.81 |
| ≥2 comorbidities | - | - | - | - | 1.01 | 0.74-1.39 | 0.90 | 0.77-1.07 | 1.00 | 0.81-1.25 | 0.56*** | 0.47-0.67 |
| **Other comorbidities** |  |  |  |  |  |  |  |  |  |  |  |  |
| Any mental health condition (yes) | - | - | 0.68** | 0.52-0.89 | - | - | - | - | - | - | 0.60*** | 0.48-0.74 |
| Tobacco use (yes) | 1.24** | 1.09-1.42 | - | - | - | - | 1.17** | 1.04-1.32 | 1.28** | 1.09-1.50 | 1.24*** | 1.10-1.34 |
| **Geographical location of residence^2^** |  |  |  |  |  |  |  |  |  |  |  |  |
| Urban | 1 |  | - |  | 1 |  | - |  | - |  | 1 |  |
| Rural | 1.64*** | 1.42-1.89 | - | - | 0.67** | 0.51-0.89 | - | - | - | - | 1.17* | 1.03-1.34 |
| **Socio-economic status^3^** |  |  |  |  |  |  |  |  |  |  |  |  |
| Most disadvantaged | 1.79*** | 1.41-2.27 | - | - | - | - | 1.30** | 1.07-1.59 | - | - | 0.72** | 0.59-0.87 |
| 2 | 1.65*** | 1.30-2.10 | - | - | - | - | 1.31** | 1.08-1.58 | - | - | 0.78** | 0.65-0.94 |
| 3 | 1.33* | 1.04-1.70 | - | - | - | - | 1.16 | 0.95-1.41 | - | - | 0.89 | 0.74-1.07 |
| 4 | 1.26 | 0.96-1.66 | - | - | - | - | 1.00 | 0.81-1.25 | - | - | 0.90 | 0.73-1.10 |
| Least disadvantaged | 1 |  | 1 |  | 1 |  | 1 |  | 1 |  | 1 |  |
| **Year of death** |  |  |  |  |  |  |  |  |  |  |  |  |
| 2014 | - |  | - |  | - |  | 1 |  | - |  | - |  |
| 2015 | - | - | - | - | - | - | 0.91 | 0.75-1.11 | - | - | - | - |
| 2016 | - | - | - | - | - | - | 0.92 | 0.76-1.12 | - | - | - | - |
| 2017 | - | - | - | - | - | - | 0.88 | 0.72-1.07 | - | - | - | - |
| 2018 | - | - | - | - | - | - | 0.79* | 0.64-0.96 | - | - | - | - |
| 2019 | - | - | - | - | - | - | 0.70** | 0.57-0.86 | - | - | - | - |
| **Hospital type at last admission** |  |  |  |  |  |  |  |  |  |  |  |  |
| Public | 1 |  | 1 |  | 1 |  | 1 |  | 1 |  | 1 |  |
| Private | 0.23*** | 0.17-0.31 | 2.23*** | 1.88-2.64 | 1.57** | 1.17-2.10 | 3.92*** | 3.41-4.51 | 2.96*** | 2.48-3.52 | 3.17*** | 2.76-3.65 |
| **History of cancer** (yes)^4^ | 1.19* | 1.02-1.39 | 1.25* | 1.05-1.48 | 1.73*** | 1.31-2.29 | 1.30*** | 1.13-1.49 | 1.32** | 1.10-1.59 | - | - |
| **Survival duration** (days) |  |  |  |  |  |  |  |  |  |  |  |  |
| 31-89 | - |  | - |  | 1 |  | 1 |  | 1 |  | 1 |  |
| ≥90 days to <180 | - | - | - | - | 0.83 | 0.53-1.29 | 0.84 | 0.64-1.11 | 0.78 | 0.53-1.14 | 0.62** | 0.47-0.83 |
| ≥180 | - | - | - | - | 0.25*** | 0.18-0.36 | 0.71** | 0.58-0.87 | 0.67** | 0.50-0.88 | 0.65*** | 0.53-0.79 |
| **Degree of cancer spread** |  |  |  |  |  |  |  |  |  |  |  |  |
| In-situ/localised | - |  | - |  | 1 |  | - |  | 1 |  | - |  |
| Regionalised | - | - | - | - | 0.99 | 0.69-1.43 | - | - | 1.05 | 0.82-1.35 | - | - |
| Metastatic | - | - | - | - | 0.53** | 0.37-0.77 | - | - | 0.77* | 0.60-0.99 | - | - |
| Not known | - | - | - | - | 0.78 | 0.48-1.25 | - | - | 0.80 | 0.57-1.11 | - | - |
| *p* < 0.001 (***), *p* < 0.01 (**), *p* ≤ 0.05 (*); AOR: Adjusted Odds Ratios.^1^ There were n=939 not known number of comorbidities excluded. ^2^ There were n=1011 not known geographical location of residence excluded ^3^ There were n=1007 not known socio-economic status excluded. ^4^ There were n=267 not known history of cancer excluded. | | | | | | | | | | | | |

Supplementary Table 6: Multivariable analysis of factors associated with potentially burdensome chemotherapy or radiotherapy indicators during 2016–2019

|  | **Last dose chemotherapy in last 7 days**  (n=194; 3.1%) | | **Last dose chemotherapy in last 14 days**  (n=388; 6.2%) | | **Radiotherapy in last 30 days**  (n=177; 2.8%) | |
| --- | --- | --- | --- | --- | --- | --- |
|  | AOR | 95% CI | AOR | 95% CI | AOR | 95% CI |
| **Age group** |  |  |  |  |  |  |
| 20–54 | 1 |  | 1 |  | 1 |  |
| 55–64 | 0.49** | 0.32-0.75 | 0.61** | 0.44-0.86 | 0.97 | 0.60-1.58 |
| 65–74 | 0.36*** | 0.24-0.54 | 0.51*** | 0.38-0.70 | 0.60* | 0.37-0.96 |
| ≥75 | 0.18*** | 0.12-0.28 | 0.22*** | 0.16-0.31 | 0.34*** | 0.21-0.53 |
| **Sex** |  |  |  |  |  |  |
| Male | - |  | 1 |  | - |  |
| Female | - | - | 0.74** | 0.59-0.92 | - | - |
| **Number of Charlson comorbidities, excluding malignancy^1^** | | | |  |  |  |
| Nil | - |  | 1 |  | - |  |
| 1 comorbidity | - | - | 0.76* | 0.60-0.96 | - | - |
| ≥2 comorbidities | - | - | 0.99 | 0.73-1.34 | - | - |
| **Other comorbidities** |  |  |  |  |  |  |
| Any mental health condition (yes) | 0.53* | 0.29-0.97 | 0.59* | 0.39-0.89 |  |  |
| Tobacco use (yes) | - | - | - | - | - | - |
| **Socio-economic status^2^** |  |  |  |  | - | - |
| Most disadvantaged | 2.49*** | 1.49-4.16 | - | - | - | - |
| 2 | 1.63 | 0.96-2.76 | - | - | - | - |
| 3 | 1.82* | 1.06-3.14 | - | - | - | - |
| 4 | 2.07* | 1.16-3.68 | - | - | - | - |
| Least disadvantaged | 1 |  | - |  | - |  |
| **Degree of cancer spread** |  |  |  |  |  |  |
| In-situ/localised | 1 |  | 1 |  | - |  |
| Regionalised | 1.07 | 0.59-1.94 | 1.50* | 0.98-2.30 | - | - |
| Metastatic | 1.86* | 1.07-3.24 | 1.96** | 1.30-2.96 | - | - |
| Not known | 1.33 | 0.65-2.69 | 1.26 | 0.74-2.15 | - | - |
| *p* < 0.001 (***), *p* < 0.01 (**), *p* ≤ 0.05 (*); AOR: Adjusted Odds Ratios.^1^ There were n=566 not known number of comorbidities excluded. ^2^There were n=615 not known socio-economic status excluded. ^3^ There were n=617 not known geographical location of residence and n=156 not known history of cancer excluded. | | | | | | |

Supplementary Table 7: Univariate and multivariable multinominal model of characteristics associated with potentially burdensome care at the end-of-life among colorectal cancer decedents

|  | **Univariate** | | | | **Multivariable** | | | |
| --- | --- | --- | --- | --- | --- | --- | --- | --- |
|  | **1 indicator** | | **≥2 indicators** | | **1 indicator** | | **≥2 indicators** | |
|  | OR | 95%CI | OR | 95%CI | AOR | 95%CI | AOR | 95%CI |
| **Age group (years)** |  |  |  |  |  |  |  |  |
| 20–54 | 1 |  | 1 |  | 1 |  | 1 |  |
| 55–64 | 0.89 | 0.72-1.11 | 0.82 | 0.64-1.06 | 0.86 | 0.69-1.08 | 0.75* | 0.57-0.98 |
| 65–74 | 0.83 | 0.68-1.00 | 0.72** | 0.57-0.91 | 0.78* | 0.64-0.96 | 0.65** | 0.51-0.83 |
| 75–84 | 0.68*** | 0.56-0.82 | 0.47*** | 0.37-0.59 | 0.66*** | 0.54-0.81 | 0.44*** | 0.35-0.57 |
| ≥85 | 0.45*** | 0.37-0.55 | 0.24*** | 0.18-0.32 | 0.48*** | 0.39-0.60 | 0.28*** | 0.21-0.37 |
| **Sex** |  |  |  |  |  |  |  |  |
| Male | 1 |  | 1 |  | 1 |  | 1 |  |
| Female | 0.84** | 0.75-0.93 | 0.71*** | 0.62-0.82 | 0.91 | 0.81-1.02 | 0.79** | 0.68-0.92 |
| **Country of birth** |  |  |  |  |  |  |  |  |
| Australia | 1.28*** | 1.14-1.44 | 1.36*** | 1.16-1.59 | 1.14* | 1.00-1.30 | 1.20* | 1.01-1.42 |
| Other/not known | 1 |  | 1 |  | 1 |  | 1 |  |
| **Number of Charlson comorbidities, excluding malignancy** | | | | | | | | |
| Nil | 1 |  | 1 |  | - | - | - | - |
| 1 comorbidity | 0.91 | 0.80-1.02 | 0.85* | 0.73-0.98 | - | - | - | - |
| ≥2 comorbidities | 0.92 | 0.79-1.07 | 0.78* | 0.64-0.95 | - | - | - | - |
| **Other comorbidities** |  |  |  |  |  |  |  |  |
| Any mental health condition (yes) | 0.81* | 0.68-0.96 | 0.66** | 0.52-0.83 | - | - | - | - |
| Tobacco use (yes) | 1.21** | 1.09-1.35 | 1.40*** | 1.22-1.60 | 1.12 | 0.99-1.25 | 1.23** | 1.09-1.43 |
| **Geographical location of residence** |  |  |  |  |  |  |  |  |
| Urban | 1 |  | 1 |  | 1 |  | 1 |  |
| Rural | 1.36*** | 1.21-1.52 | 1.36*** | 1.18-1.57 | 1.21** | 1.07-1.38 | 1.24* | 1.05-1.46 |
| **Socio-economic status** |  |  |  |  |  |  |  |  |
| Most disadvantaged | 1.20* | 1.05-1.48 | 1.20 | 0.97-1.50 | 1.34** | 1.11-1.61 | 1.31* | 1.03-1.67 |
| 2 | 1.23** | 1.12-1.56 | 1.23 | 0.99-1.52 | 1.34** | 1.12-1.61 | 1.28* | 1.01-1.63 |
| 3 | 1.05 | 0.88-1.26 | 1.06 | 0.85-1.34 | 1.10 | 0.91-1.32 | 1.08 | 0.85-1.38 |
| 4 | 0.91 | 0.74-1.11 | 0.88 | 0.68-1.15 | 0.98 | 0.79-1.21 | 0.96 | 0.72-1.26 |
| Least disadvantaged | 1 |  | 1 |  | 1 |  | 1 |  |
| **Year of death** |  |  |  |  |  |  |  |  |
| 2014 | 1 |  | 1 |  | - |  | - |  |
| 2015 | 1.02 | 0.85-1.22 | 1.01 | 0.79-1.28 | - | - | - | - |
| 2016 | 1.01 | 0.85-1.21 | 1.04 | 0.82-1.32 | - | - | - | - |
| 2017 | 0.99 | 0.82-1.18 | 1.01 | 0.80-1.28 | - | - | - | - |
| 2018 | 1.06 | 0.88-1.27 | 1.06 | 0.83-1.34 | - | - | - | - |
| 2019 | 0.96 | 0.80-1.15 | 0.99 | 0.78-1.26 | - | - | - | - |
| **Hospital type at last admission** |  |  |  |  |  |  |  |  |
| Public | 1 |  | 1 |  | 1 |  | 1 |  |
| Private | 1.93*** | 1.68-2.21 | 2.16*** | 1.82-2.56 | 2.06*** | 1.78-2.39 | 2.26*** | 1.88-2.72 |
| **History of cancer** (yes) | 1.10 | 0.97-1.24 | 1.15 | 0.99-1.35 | 1.24** | 1.09-1.41 | 1.43*** | 1.21-1.69 |
| **Survival duration** (days) |  |  |  |  |  |  |  |  |
| 31–89 | 1 |  | 1 |  | 1 |  | 1 |  |
| ≥90 days to <180 | 0.85 | 0.65-1.12 | 1.33 | 0.95-1.87 | 0.85 | 0.64-1.11 | 1.31 | 0.93-1.85 |
| ≥180 | 0.82* | 0.68-1.00 | 0.89 | 0.68-1.16 | 0.81* | 0.66-0.99 | 0.84 | 0.64-1.10 |
| **Degree of cancer spread** |  |  |  |  |  |  |  |  |
| In-situ/localised | 1 |  | 1 |  | - |  | - |  |
| Regionalised | 1.14 | 0.97-1.35 | 1.11 | 0.88-1.39 | - | - | - | - |
| Metastatic | 1.21* | 1.02-1.42 | 1.33* | 1.06-1.65 | - | - | - | - |
| Not known | 1.03 | 0.84-1.26 | 1.16 | 0.88-1.51 | - | - | - | - |
| *p* < 0.001 (***), *p* < 0.01 (**), *p* ≤ 0.05 (*); OR: Unadjusted Odds Ratios; AOR: Adjusted Odds Ratios | | | | | | | | |
